# Supplementary material for: PRC1 collaborates with SMCHD1 to fold the X-chromosome and spread Xist RNA between chromosome compartments
Source: Nat Commun. 2019 Jul 3;10:2950. doi: 10.1038/s41467-019-10755-3 (PMC6610634; doi:10.1038/s41467-019-10755-3)
Supplement: Supplementary file 1 — Supplementary Information [file 41467_2019_10755_MOESM1_ESM.pdf]

Supplementary Information

PRC1 collaborates with SMCHD1 to fold the X-chromosome and spread Xist RNA between chromosome compartments

Wang *et al*.

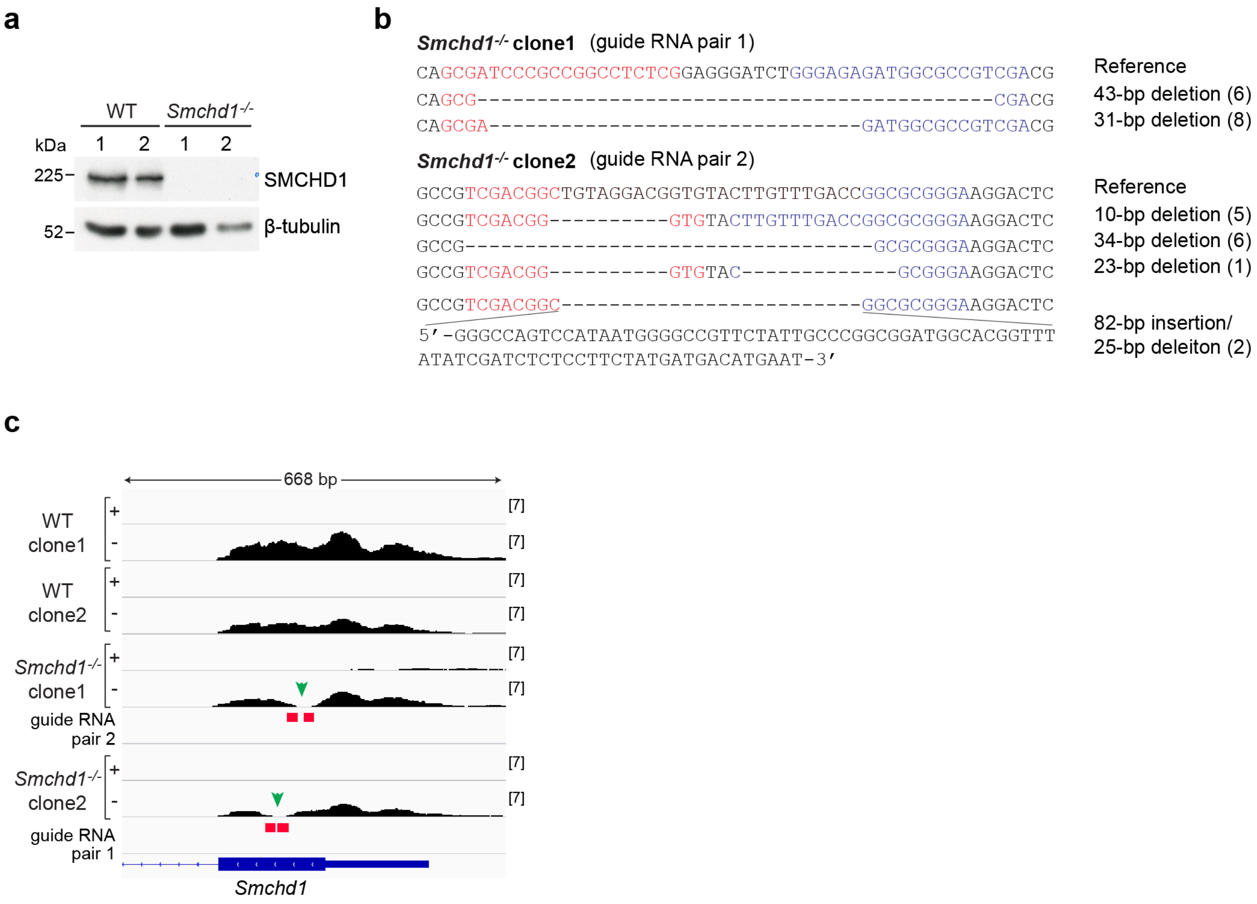

Supplementary Figure 1. A model to study the role of SMCHD1 in the maintenance phase of XCI. **(a)** Western blots confirmed the absence of SMCHD1 protein in *Smchd1*$^{-/-}$ clones. Shown are two wild-type clones (WT clone 1 and 2) and two *Smchd1*$^{-/-}$ clones (*Smchd1*$^{-/-}$ clone 1 and 2). WT clones were generated by transfecting the parental line with plasmids expressing Cas9 nickase but not guide RNAs. **(b)** Sanger sequencing confirmed the presence of frame-shifting mutations at *Smchd1* loci in *Smchd1*$^{-/-}$ clones. The regions encompassing CRISPR-target sites were PCR amplified and cloned into a TOPO-TA vector for sequencing. Blue & red: sequences targeted by guide RNAs. Numbers within parentheses, the number of clones sequenced. Note that this MEF line is tetraploid (4n) due to SV-40 large T transformation. Therefore, one *Smchd1*$^{-/-}$ clone may harbor >2 different mutant alleles. *Smchd1*$^{-/-}$ clone 2 carries an allele where 25 nucleotides were replaced with an 87-nucleotide insertion. The inserted sequences were also listed. **(c)** RNA-seq coverage tracks at the exon 1 of the *Smchd1* locus showed evident microdeletions in *Smchd1*$^{-/-}$ clones. Note that *Smchd1*$^{-/-}$ clone 1 and 2 harbored distinct microdeletions as they were generated independently by two different pairs of guide RNAs. +, the plus strand. -, the minus strand.

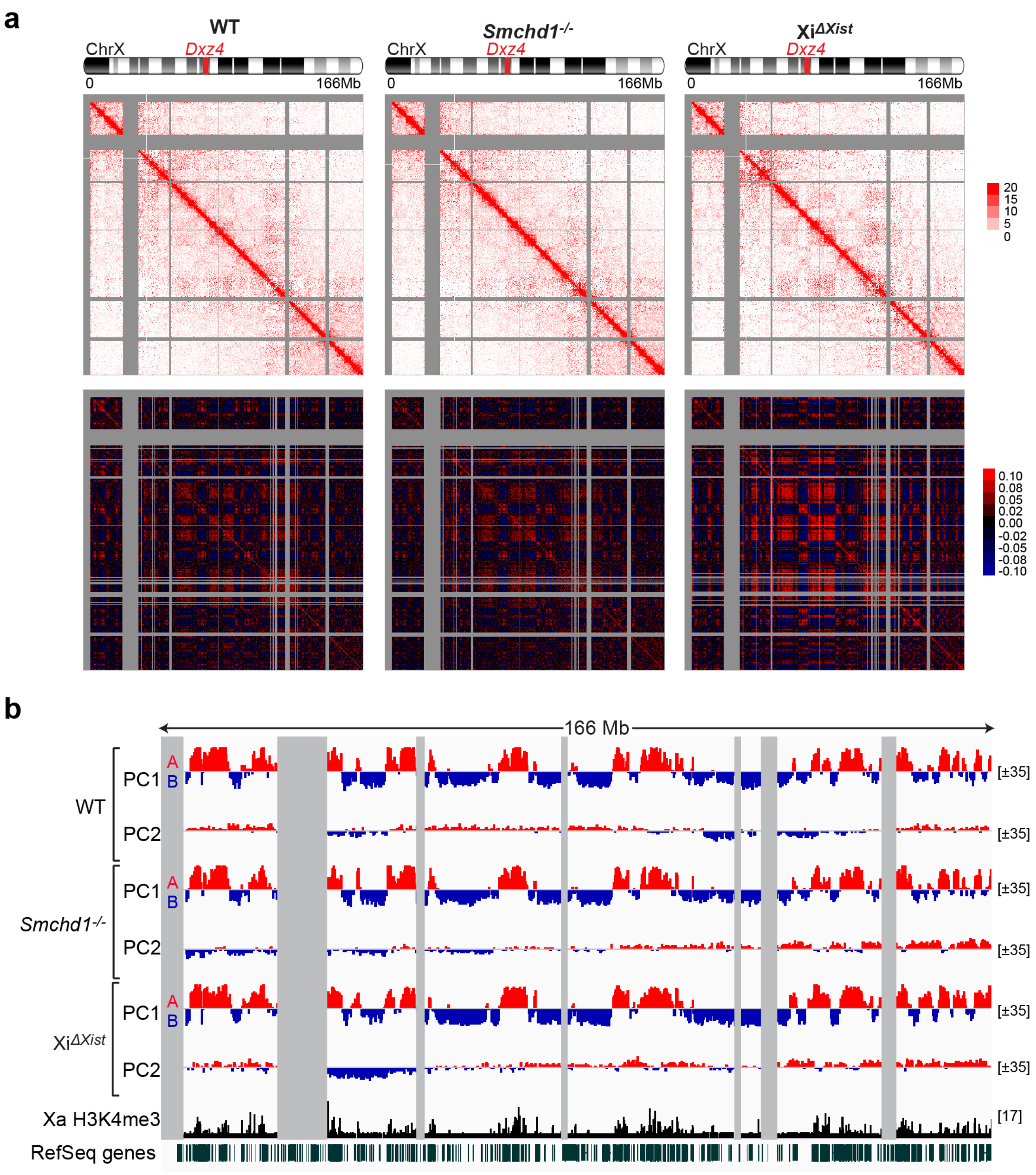

Supplementary Figure 2. The A/B compartments on the Xa are unaffected by *Smchd1* or *Xist* ablation. (a) Depth-corrected chromatin interaction maps of the Xa in WT, *Smchd1*$^{-/-}$, and Xi$^{\Delta Xist}$ fibroblasts binned at 200-kb resolution (top) and the corresponding Pearson correlation maps (bottom). Gray-shaded areas, unmappable regions. We noted that the intensity of the Pearson correlation maps is influenced by the sequencing depth of Hi-C libraries. To facilitate visual comparison of the Pearson correlation maps under different treatment conditions, we displayed depth-corrected Hi-C contact maps and Pearson correlation maps, which were generated by down-sampling Hi-C data randomly. **(b)** PC1 and PC2 values of the Xa. Gray-shaded areas, unmappable regions.

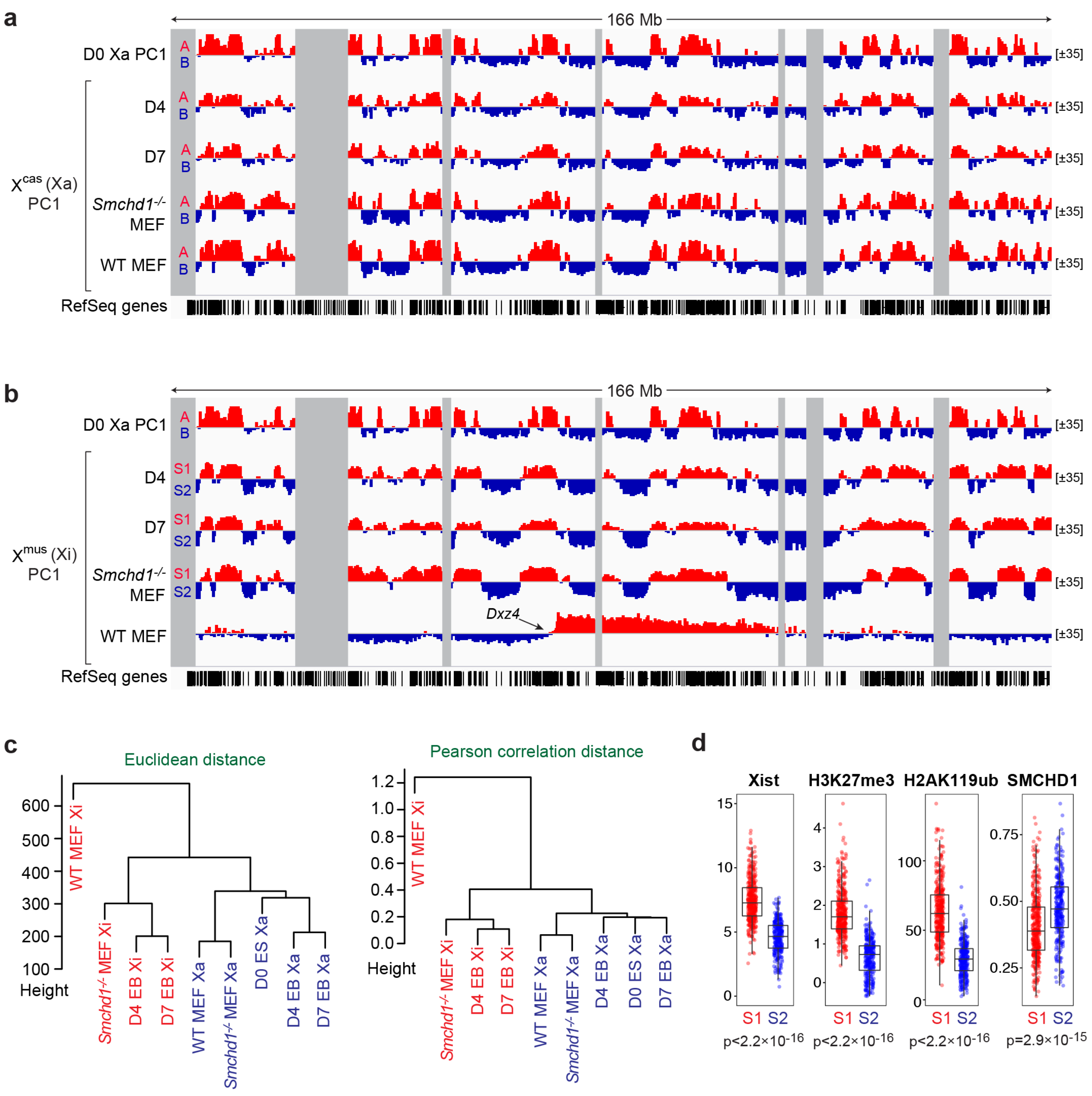

Supplementary Figure 3. S1/S2 compartments reappear upon post-XCI *Smchd1* ablation. (a) PC1 values of the Xa in undifferentiated wild-type (WT) female embryonic stem (ES) cells (D0 ES, pre-XCI), embryoid bodies (EB) formed after 4 (D4) days of differentiation (D4 EB, early XCI), D7 EB, *Smchd1*$^{-/-}$, and WT MEFs. Regions with positive PC1 values represent the A (active) compartment (red). Gray-shaded areas, unmappable regions. ES and EB data were from GSE99991. **(b)** PC1 values of the Xa in D0 ES cells and the Xi in D4 EB, D7 EB, *Smchd1*$^{-/-}$, and WT MEFs. Regions with positive PC1 values in D4 EB, D7 EB, and *Smchd1*$^{-/-}$ MEFs represent the S1 (Xist-rich) compartment (red). **(c)** Hierarchical clustering analysis using the Euclidean distance (left) or the Pearson correlation distance (right) of PC1 profiles on the Xa and Xi in D0 ES, D4 EB, D7 EB, *Smchd1*$^{-/-}$, and WT MEFs. **(d)** Box plots comparing the

density of Xist (CHART-seq, GSE48649), H3K27me3 (ChIP-seq, GSE33823), H2AK119ub (ChIP-seq, GSE107217), and SMCHD1 (DamID-seq, GSE99991) of each 200-kb bin in S1 versus S2 compartments. *P*-values given by the Wilcoxon rank sum test (unpaired, one-sided). Midline, median. Top and bottom of the box, first and third quartile. Whiskers, extension from the top or bottom to the furthest datum within 1.5 times the interquartile range.

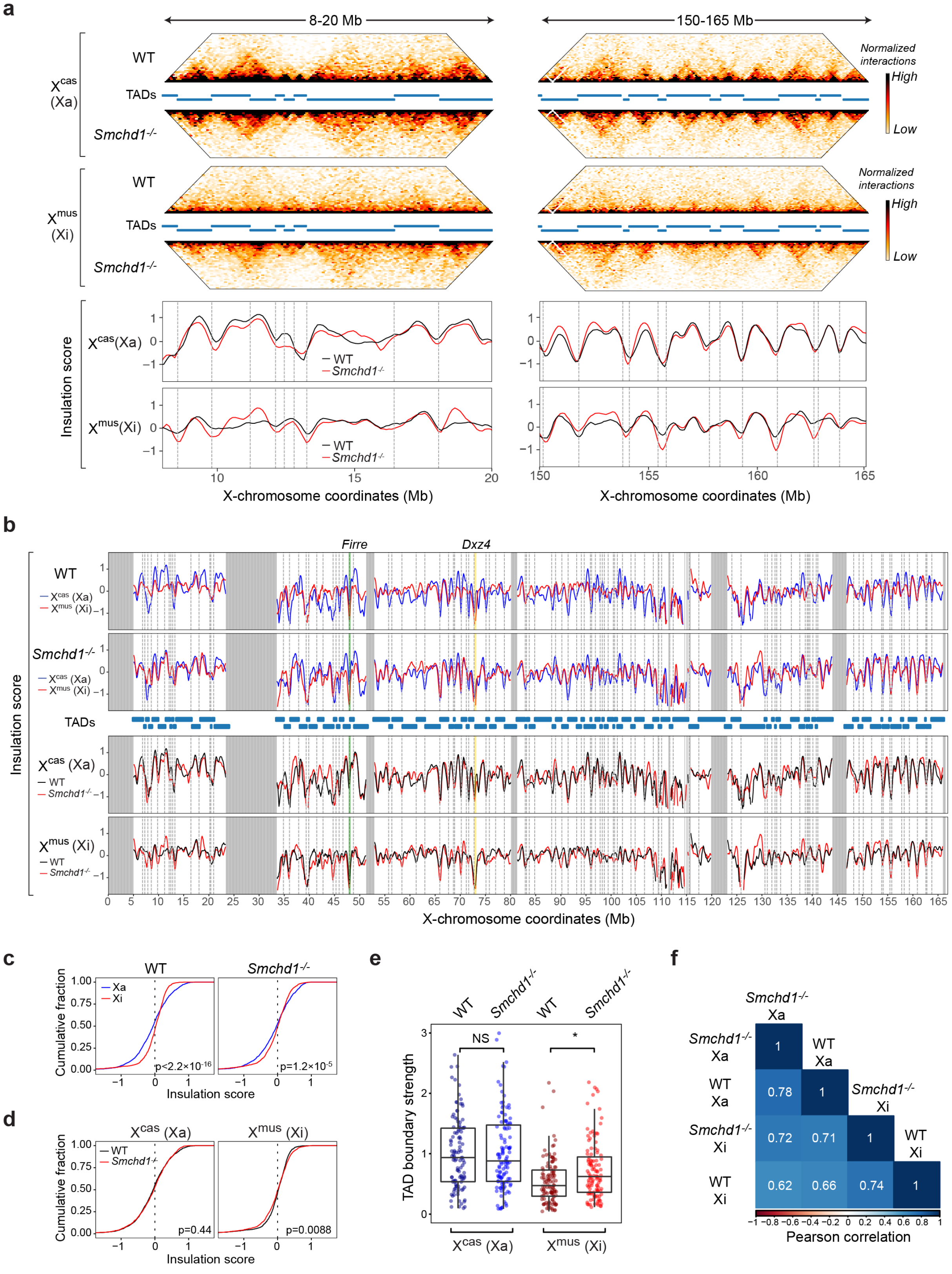

Supplementary Figure 4. Ablating *Smchd1* partially restores TADs on the Xi in post-XCI cells. (a)

Hi-C interaction maps (binned at 100-kb resolution) and insulation profiles at two representative regions on the X. TADs (as defined in Dixon *et al*., 2012 [1]) were depicted as blue bars between interaction maps and as dashed lines in insulation plots. **(b)** Pair-wise comparison of the insulation profiles of the Xa and the Xi in wild-type (WT) and *Smchd1*$^{-/-}$ MEFs. TADs (as defined in Dixon *et al*., 2012 [1]) were depicted as blue bars between plots and as dashed lines within each plot. Green-shaded area, *Firre*. Yellow-shaded area, *Dxz4*. Gray-shaded areas, unmappable regions. **(c)** Cumulative distribution plots (CDP) of the insulation scores of each 100-kb bin on the Xa (blue) and the Xi (red) in WT (left) and *Smchd1*$^{-/-}$ (right) MEFs. *P*-values given by the Kolmogorov–Smirnov (KS) test. **(d)** CDPs of the insulation scores between WT (black) and *Smchd1*$^{-/-}$ (red) MEFs. Xa, left. Xi, right. *P*-values given by the KS test. **(e)** Box plots comparing the boundary strength (defined as the differences in insulation scores between insulation minimum to the largest neighboring insulation maximum) of the Xa and the Xi in WT and *Smchd1*$^{-/-}$ MEFs. *P*-values given by the Wilcoxon ranked sum test (unpaired, two-sided). NS, not significant (p>0.05). *, p=0.0035. Midline, median. Top and bottom of the box, first and third quartile. Whiskers, extension from the top or bottom to the furthest datum within 1.5 times the interquartile range. **(f)** Correlation analysis of the insulation profiles presented in (b), (c), and (d).

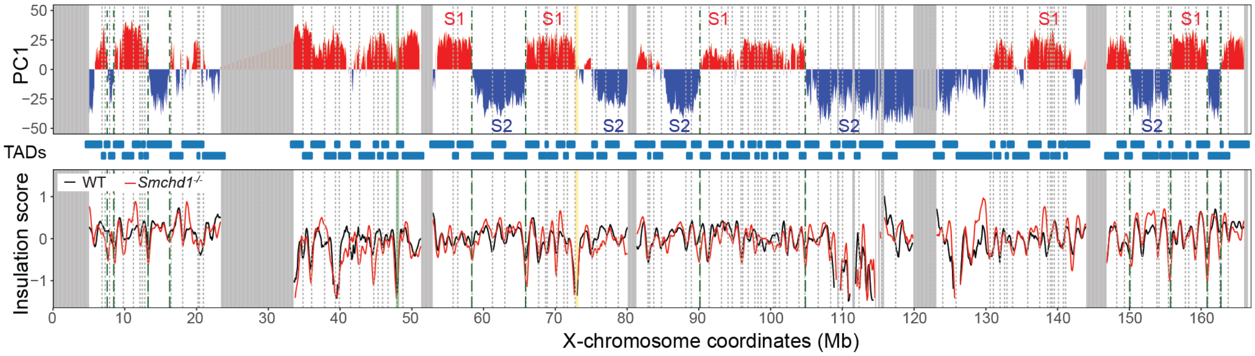

Supplementary Figure 5. Borders of S1/S2 compartments often coincide with TAD boundaries. Chromosome-wide comparison of the compartment profiles (PC1) (top) of the *Smchd1*$^{-/-}$ Xi and the insulation profiles (bottom) of the Xi in wild-type (WT, black) and *Smchd1*$^{-/-}$ (red) MEFs. TADs (as defined in Dixon *et al*., 2012 [1]) were depicted as blue bars between plots and as dashed lines within each plot. Green dashed lines, the borders of S1/S2 compartments. Green-shaded area, *Firre*. Yellow-shaded area, *Dxz4*. Gray-shaded areas, unmappable regions.

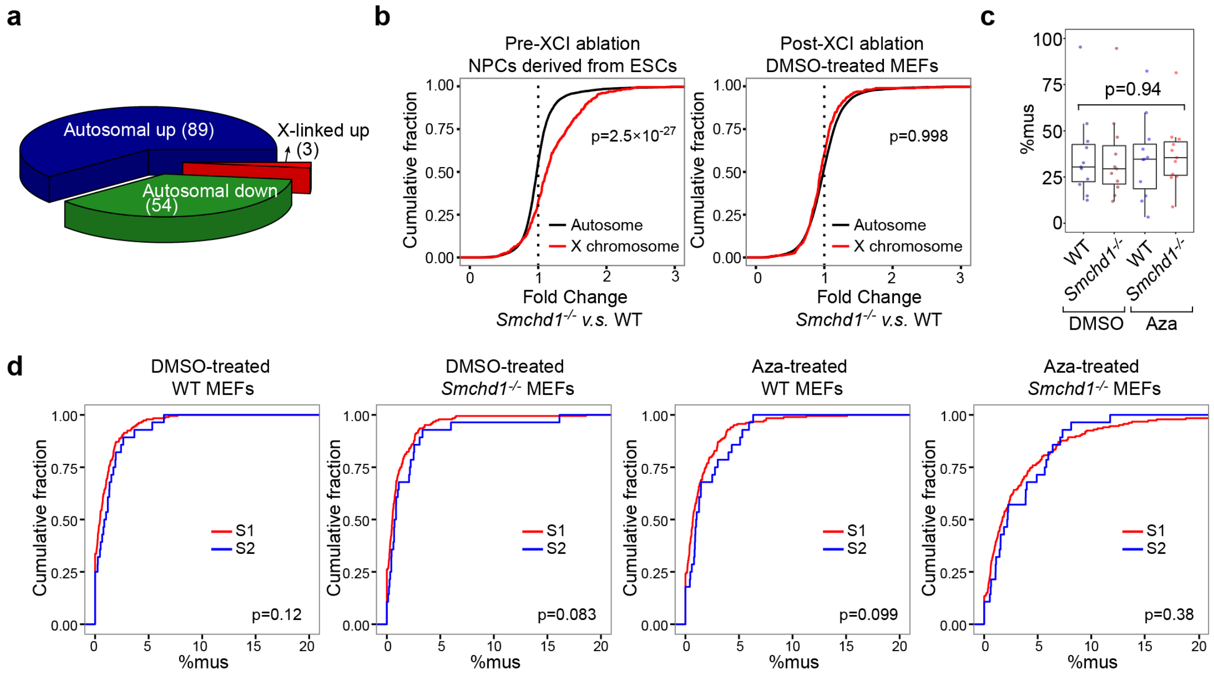

Supplementary Figure 6. Post-XCI ablation of *Smchd1* does not cause upregulation of X-linked genes. (a) A pie chart showing the chromosomal distribution of differentially expressed genes. Autosomal up: upregulated autosomal genes. Autosomal down, downregulated autosomal genes. X-linked up, upregulated X-linked genes. Numbers within parentheses indicate the numbers of genes in each category. **(b)** Cumulative distribution plots (CDPs) comparing fold-changes of expressed X-linked genes (red) versus autosomal genes (black) between neural progenitor cells (NPCs) derived from female WT and *Smchd1*$^{-/-}$ mouse embryonic stem (ES) cells (pre-XCI ablation, left)(GSE99991), or between female WT and *Smchd1*$^{-/-}$ MEFs treated with DMSO (post-XCI ablation, right). *P*-values given by the Wilcoxon ranked sum test (unpaired, one-sided). Note that in contrast to cells undergoing XCI in the absence of SMCHD1 protein (pre-XCI *Smchd1* ablation), losing SMCHD1 in cells that have already completed XCI (post-XCI *Smchd1* ablation) does not lead to detectable upregulation of X-linked genes. **(c)** Box plots showing the %mus of escapees. *P*-values given by Kruskal-Wallis rank sum test. Please see Supplementary Data 1 for the full list of escapees. Note that here we analyzed 11 genes that met the definition of escapees in the RNA-seq data of both WT MEF clones, whereas in Fig. 2b, 14 genes were listed as escapees because they met the definition of escapees in at least one WT MEF clone. Midline, median. Top and bottom of the box, first and third quartile. Whiskers, extension from the top or bottom to the furthest datum within 1.5 times the interquartile range. **(d)** CDPs comparing %mus of genes subject to the Xi in S1 versus S2 compartments. *P*-values given by the Wilcoxon rank sum test (unpaired, one-sided).

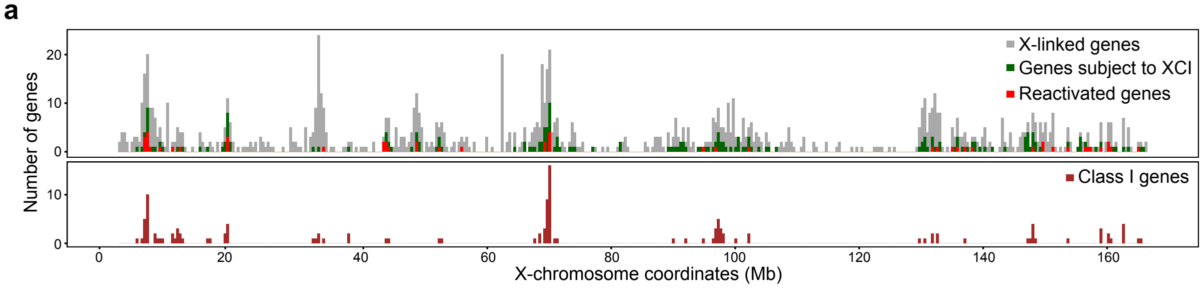

b

Class	Total	Reactivated	%	p-value
Class I	79	30	38	0.00046
Class II	42	10	24	1
Class III	73	8	11	0.012
Unclassified	19	4	21	1
Silent	2	0	0	1
Escapees	1	0	0	1
Total	216	52	24	

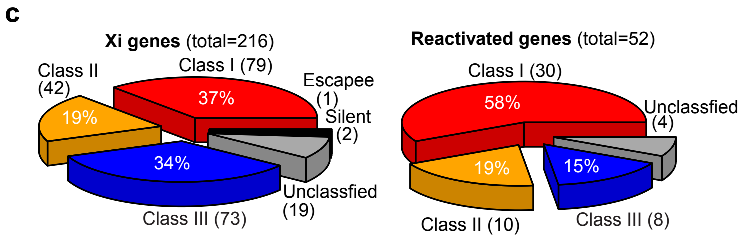

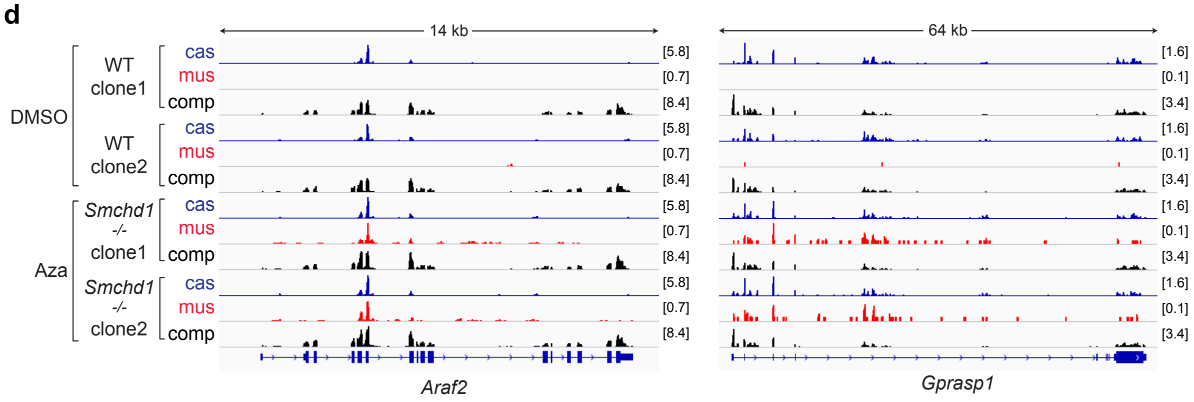

Supplementary Figure 7. Class I genes are more sensitive to combined *Smchd1* ablation and Aza treatment. (a) X-chromosomal locations of genes reactivated by dual *Smchd1* ablation and Aza treatment (reactivated genes). Also shown are locations of all X-linked genes, genes subject to XCI, and Class I genes (defined in Wang *et al.*, 2018 [2], also see Methods). The occurrence of reactivated genes shows mild positive correlation with Class I genes (*r*=0.43). **(b)** A table showing the distribution of X-linked gene classes defined previously in mouse neural progenitor cells (NPCs) [2] within the Xi genes and reactivated genes in this study. %, fraction of reactivated genes within each category. *P*-values given by the Fisher's exact test (two-sided). Note that among 126 Class I genes reported previously, 79 genes passed our pipeline requirement for allele-specific analysis in this study. Please see Supplementary Data 2 for the full list of genes in each X-linked category. **(c)** Pie charts displaying the categorization of Xi genes and reactivated genes. Number of genes in each category shown in parenthesis. Note that one gene subject to XCI in MEFs was defined as an escapee in NPCs. **(d)** Allele-specific RNA-seq coverage tracks of two Class I genes. cas tracks, cas-specific reads (Xa). mus, mus-specific reads (Xi). comp, all reads. To visualize rare mus reads originating from the Xi, the scales of mus tracks were set differently from the cas tracks. For simplicity, only the plus strand was shown.

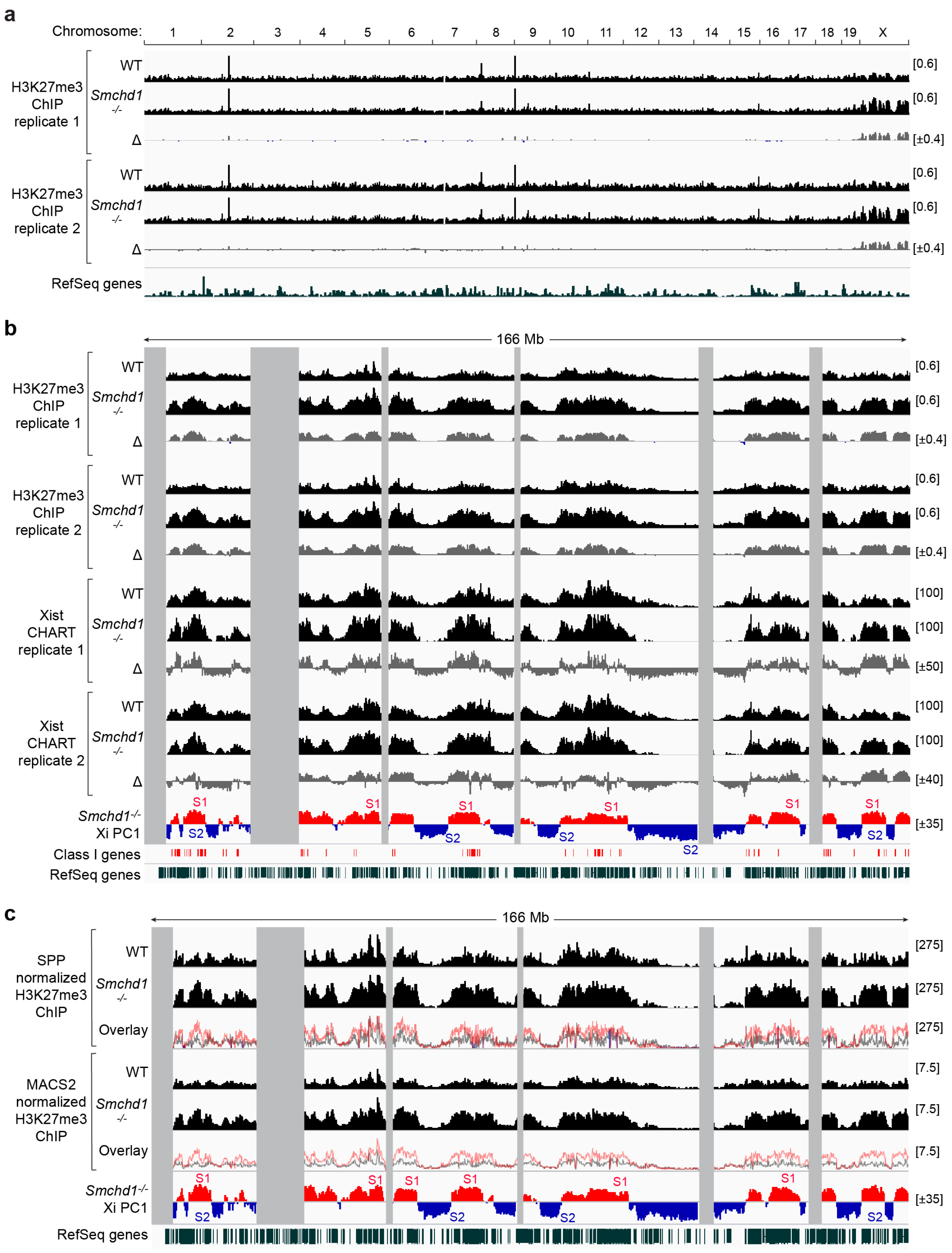

Supplementary Figure 8. Resurrected S1/S2 compartments cause aberrant distribution of H3K27me3 and Xist. (a) H3K27me3 enrichment profiles across the entire genome. Two biological

replicates shown. Δ, *Smchd1*$^{-/-}$ minus WT. **(b)** H3K27me3 and Xist enrichment profiles across the X chromosome. Gray areas, unmappable regions. Also shown are the locations of Class I genes (red bars), and S1/S2 compartments in *Smchd1*$^{-/-}$ MEFs. Two biological replicates shown. **(c)** H3K27me3 enrichment profiles computed by two different methods of ChIP-seq normalization across the X chromosome. In the two “Overlay” tracks, the black line represents the WT profile, and the red line represents the *Smchd1*$^{-/-}$ profile.

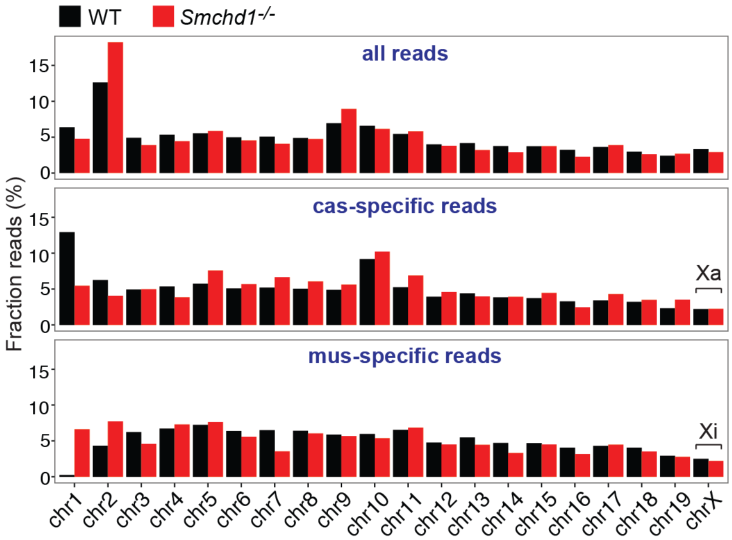

Supplementary Figure 9. Wild-type and *Smchd1*$^{-/-}$ MEFs have similar copy number of the X chromosomes. Histograms comparing the distribution of reads mapped to each chromosome in the ChIP-seq input datasets between wild-type (WT) and *Smchd1*$^{-/-}$ MEFs. Note that despite some changes in autosomes, the Xs exhibit similar fraction mapped reads between WT and *Smchd1*$^{-/-}$ MEFs.

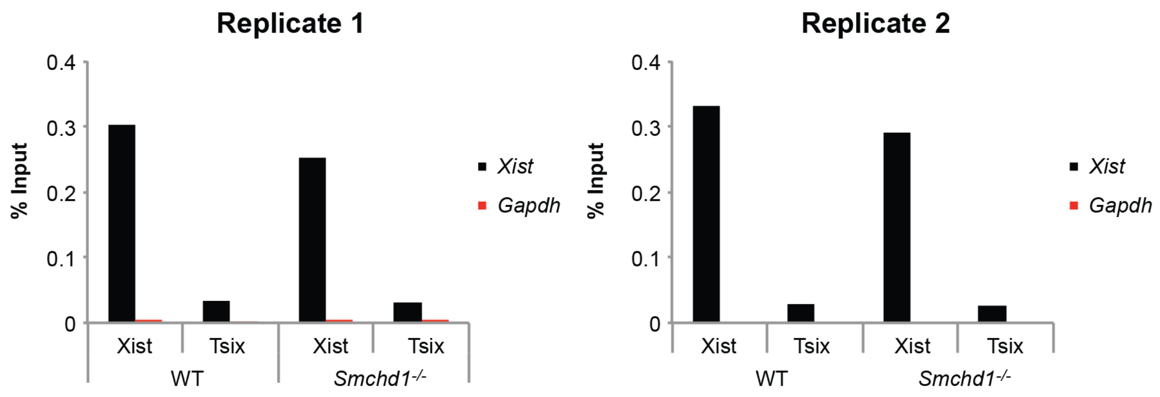

Supplementary Figure 10. Quality control for CHART experiments in wild-type (WT) and *Smchd1*$^{-/-}$ MEFs. CHART-qPCR analysis for the promoter of *Xist* (positive control) and *Gapdh* (autosomal negative control). Also shown are control CHART experiments using negative control probes antisense to the Xist capturing probes (labeled as "Tsix"). The same batch of CHART-enriched DNA was then subject to library preparation and deep sequencing. Two biological replicates shown.

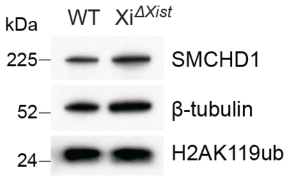

Supplementary Figure 11. *Xist* deletion on the Xi does not decrease the overall level of the SMCHD1 protein and the H2AK119ub mark. Western blot showing that SMCHD1 and H2AK119ub remained detectable in Xi$^{\Delta Xist}$ fibroblasts, indicating that the absence of their enrichment on the Xi is not caused by global reduction of SMCHD1 and H2AK119ub.

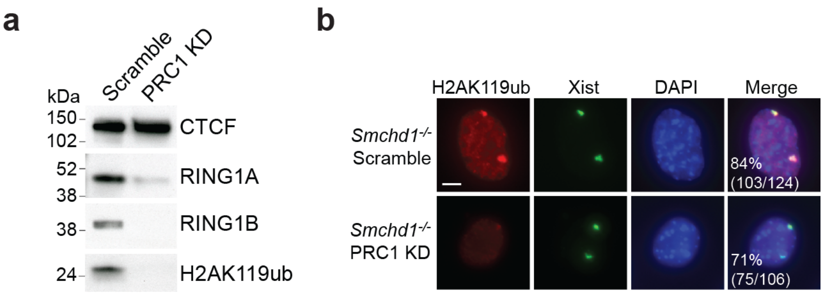

Supplementary Figure 12. Efficient depletion of PRC1 in *Smchd1*$^{-/-}$ MEFs. (a) Western blot confirming efficient knockdown of RING1A and RING1B ("PRC1 KD") and the resulting H2AK119ub depletion in *Smchd1*$^{-/-}$ MEFs used for Hi-C (Fig. 6). CTCF, loading control. **(b)** Immuno-RNA-FISH for H2AK119ub and Xist on *Smchd1*$^{-/-}$ MEFs treated with scramble or RING1A/RING1B (PRC1 KD) siRNA. Number of cells with the presented pattern (scramble: Xist clouds with colocalizing H2AK119ub foci; PRC1 KD: Xist clouds with attenuated H2AK119ub signals) shown. Scale bar, 5μm.

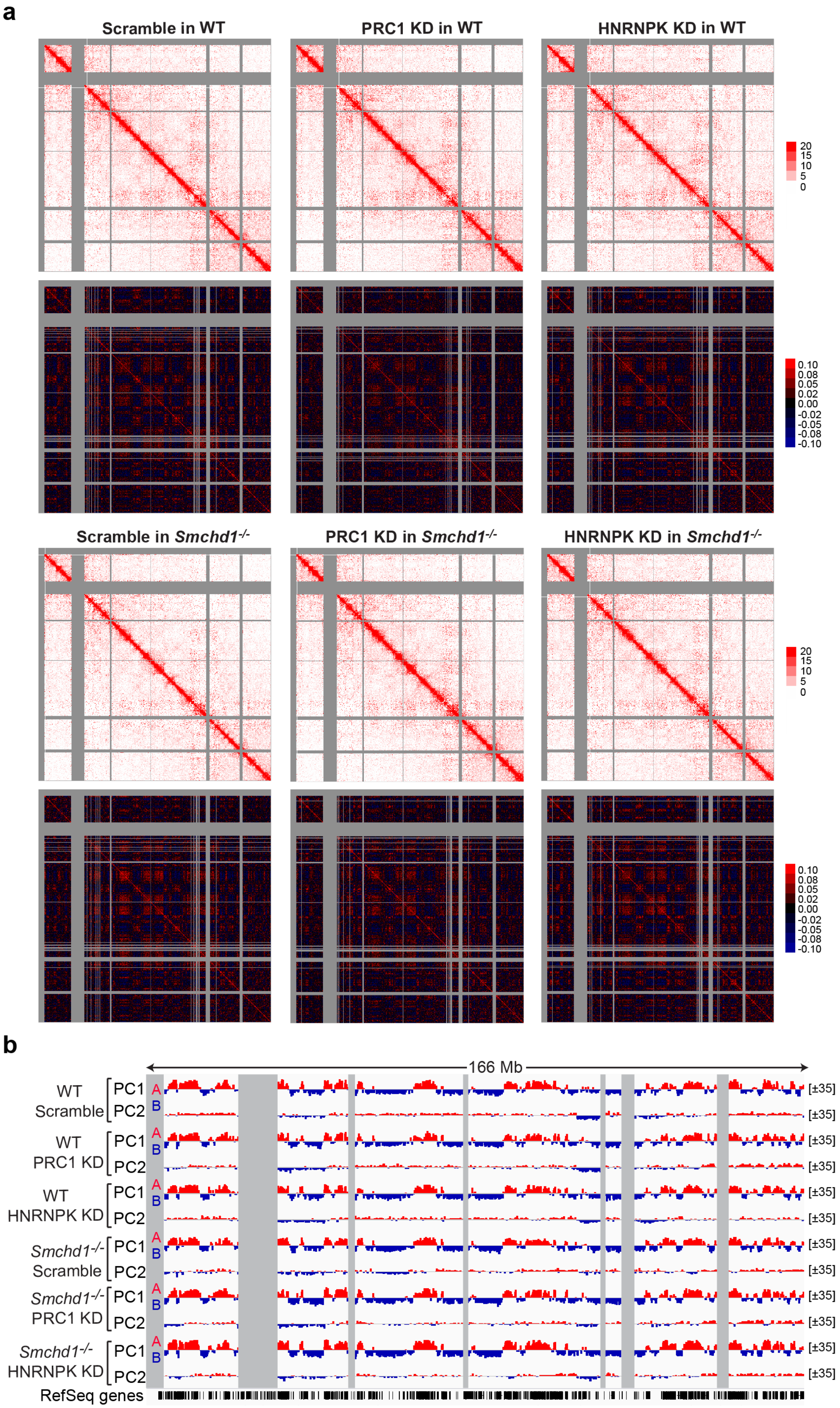

Supplementary Figure 13. The A/B compartments on the Xa appear unaffected by PRC1 or

HNRNPK depletion. (a) Depth-corrected chromatin interaction maps of the Xa in WT or *Smchd1*$^{-/-}$ MEFs treated with control (Scramble), RING1A/RING1B (PRC1 KD), or HNRNPK (HNRNPK KD) siRNA binned at 200-kb resolution (top) and the corresponding Pearson correlation maps (bottom). Gray-shaded areas, unmappable regions. **(b)** PC1 and PC2 values of the Xa. Gray-shaded areas, unmappable regions.

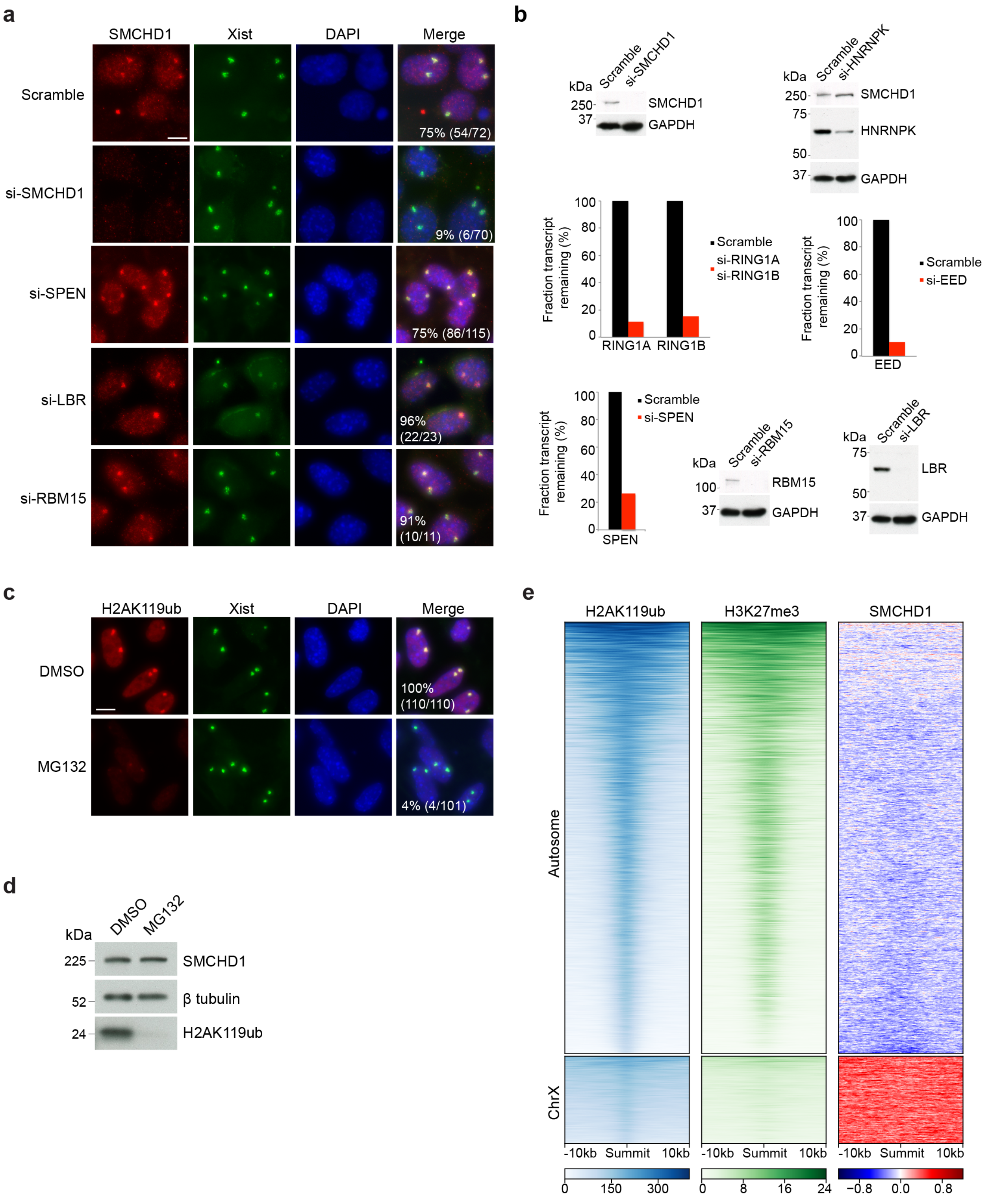

Supplementary Figure 14. HNRNPK and PRC1 facilitate SMCHD1 recruitment to the Xi. (a) Immuno-RNA-FISH for SMCHD1 and Xist on female MEFs treated with siRNAs targeting SMCHD1 and three

candidate Xist-interacting proteins. Number of cells with SMCHD1 foci co-localizing with Xist clouds in a representative biological replicate shown. Scale bar, 10μm. **(b)** Western blot and RT-qPCR confirming efficient knockdown of indicated proteins in cells used for immuno-RNA-FISH. GAPDH, loading control. Note that the level of SMCHD1 protein was not affected by HNRNPK knockdown, indicating that the absence of SMCHD1 enrichment on the Xi is not caused by global reduction of SMCHD1 protein. **(c)** Immuno-RNA-FISH for H2AK119ub and Xist on female MEFs treated with DMSO or MG132. Number of DMSO-treated cells with H2AK119ub foci co-localizing with Xist clouds shown. For MG132-treated cells, nuclei exhibiting H2AK119ub signals with intensity similar to that of DMSO-treated cells shown. Scale bar, 10μm. **(d)** Western blot confirming efficient depletion of H2AK119ub in cells treated with MG132. β-tubulin, loading control. Note that the level of SMCHD1 protein was not affected by MG132 treatment, indicating that the absence of SMCHD1 enrichment on the Xi is not caused by global reduction of SMCHD1 protein. **(e)** Heatmaps displaying the distribution of H2AK119ub (GSE107217), H3K27me3 (GSE33823), and SMCHD1 (GSE99991) over each 20-kb region centered at the summit of an H2AK119ub peak. Note the SMCHD1-binding profiles were determined by DamID and presented as the log2 ratio of Dam-SMCHD1 over Dam alone control. Thus, a negative value in the SMCHD1 profiles indicates that SMCHD1 binding is depleted relative to the Dam alone control.

Supplementary References

1 Dixon, J. R., Selvaraj, S., Yue, F., Kim, A., Li, Y., Shen, Y., Hu, M., Liu, J. S. & Ren, B. Topological domains in mammalian genomes identified by analysis of chromatin interactions. *Nature* **485**, 376-380 (2012).

2 Wang, C. Y., Jegu, T., Chu, H. P., Oh, H. J. & Lee, J. T. SMCHD1 Merges Chromosome Compartments and Assists Formation of Super-Structures on the Inactive X. *Cell* (2018).
