# Supplementary material for: PRC1 collaborates with SMCHD1 to fold the X-chromosome and spread Xist RNA between chromosome compartments
Source: Nat Commun. 2019 Jul 3;10:2950. doi: 10.1038/s41467-019-10755-3 (PMC6610634; doi:10.1038/s41467-019-10755-3)
Supplement: Supplementary file 3 — Description of Additional Supplementary Files [file 41467_2019_10755_MOESM3_ESM.pdf]

Description of Additional Supplementary Files

File name: Supplementary Data 1

Description: X-linked gene classification in MEFs

File name: Supplementary Data 2

Description: X-linked gene classification in Wang et al., 2018, Cell

File name: Supplementary Data 3

Description: Oligonucleotides used in this study
