# Supplementary material for: PRC1 collaborates with SMCHD1 to fold the X-chromosome and spread Xist RNA between chromosome compartments
Source: Nat Commun. 2019 Jul 3;10:2950. doi: 10.1038/s41467-019-10755-3 (PMC6610634; doi:10.1038/s41467-019-10755-3)
Supplement: Supplementary file 7 — Reporting Summary [file 41467_2019_10755_MOESM7_ESM.pdf]

nature research

Corresponding author(s): Jeannie T. Lee

Reporting Summary

Nature Research wishes to improve the reproducibility of the work that we publish. This form provides structure for consistency and transparency in reporting. For further information on Nature Research policies, see Authors & Referees and the Editorial Policy Checklist.

Statistical parameters

When statistical analyses are reported, confirm that the following items are present in the relevant location (e.g. figure legend, table legend, main text, or Methods section).

n/a	Confirmed	
☐	☒	The exact sample size (n) for each experimental group/condition, given as a discrete number and unit of measurement
☐	☒	An indication of whether measurements were taken from distinct samples or whether the same sample was measured repeatedly
☐	☒	The statistical test(s) used AND whether they are one- or two-sided<br>*Only common tests should be described solely by name; describe more complex techniques in the Methods section.*
☒	☐	A description of all covariates tested
☒	☐	A description of any assumptions or corrections, such as tests of normality and adjustment for multiple comparisons
☒	☐	A full description of the statistics including central tendency (e.g. means) or other basic estimates (e.g. regression coefficient) AND variation (e.g. standard deviation) or associated estimates of uncertainty (e.g. confidence intervals)
☐	☒	For null hypothesis testing, the test statistic (e.g. F, t, r) with confidence intervals, effect sizes, degrees of freedom and P value noted<br>*Give P values as exact values whenever suitable.*
☒	☐	For Bayesian analysis, information on the choice of priors and Markov chain Monte Carlo settings
☒	☐	For hierarchical and complex designs, identification of the appropriate level for tests and full reporting of outcomes
☒	☐	Estimates of effect sizes (e.g. Cohen's d, Pearson's r), indicating how they were calculated
☐	☒	Clearly defined error bars<br>*State explicitly what error bars represent (e.g. SD, SE, CI)*

Our web collection on statistics for biologists may be useful.

Software and code

Policy information about availability of computer code

Data collection	Images were acquired with a Nikon Eclipse 90i microscope and a Hamamatsu CCD camera. Image analysis was performed using Volocity (Perkin-Elmer).
Data analysis	Software used has been decribed in "Methods." Please also find a list below:<br>Trim Galore!<br>novoalign/novoalign_v3-1.00.02<br>samtools0118<br>SPP<br>homer/4.8<br>HiTC<br>cutadapt/1.4.2<br>deeptools/2.2.4<br>tophat/2.0.10

For manuscripts utilizing custom algorithms or software that are central to the research but not yet described in published literature, software must be made available to editors/reviewers upon request. We strongly encourage code deposition in a community repository (e.g. GitHub). See the Nature Research guidelines for submitting code & software for further information.

Data

Policy information about availability of data

All manuscripts must include a data availability statement. This statement should provide the following information, where applicable:
- Accession codes, unique identifiers, or web links for publicly available datasets
- A list of figures that have associated raw data
- A description of any restrictions on data availability

All NGS data (raw, processed, and re-analyzed) have been uploaded to the Gene Expression Omnibus, under GSE116413.

Field-specific reporting

Please select the best fit for your research. If you are not sure, read the appropriate sections before making your selection.

☒ Life sciences ☐ Behavioural & social sciences ☐ Ecological, evolutionary & environmental sciences

For a reference copy of the document with all sections, see nature.com/authors/policies/ReportingSummary-flat.pdf

Life sciences study design

All studies must disclose on these points even when the disclosure is negative.

Sample size	RNA-seq was performed on two independently derived Smchd1-/- MEF clones and two wild-type MEF clones. H3K27me3 ChIP-seq, H2AK119ub ChIP-seq, Xist CHART-seq, and Hi-C were performed on one Smchd1-/- and one wild-type clone. In immuno-RNA-FISH experiments, the number of cells counted was listed in each figure.
Data exclusions	No data exclusions.
Replication	RNA-seq was performed in two wild-type and two Smchd1-/- clones. H3K27me3 ChIP-seq and Xist CHART-seq were performed in two biological replicates. H2AK119ub ChIP-seq was performed in one biological replicate. All Hi-C was performed in two biological replicates, except for Hi-C on PRC1-depleted WT cells, which was performed once. Immuno-RNA-FISH was performed with at least two biological replicates.
Randomization	Not applicable.
Blinding	Not applicable.

Reporting for specific materials, systems and methods

Materials & experimental systems

n/a	Involved in the study
☐	☒ Unique biological materials
☐	☒ Antibodies
☐	☒ Eukaryotic cell lines
☒	☐ Palaeontology
☒	☐ Animals and other organisms
☒	☐ Human research participants

Methods

n/a	Involved in the study
☐	☒ ChIP-seq
☒	☐ Flow cytometry
☒	☐ MRI-based neuroimaging

Unique biological materials

Policy information about availability of materials

Obtaining unique materials	All unique biological materials will be available upon request.

Antibodies

Antibodies used	H3K27me3 (GTX60892, GENETEX)<br>H3K27me3 (07-449, Millipore)

H2AK119ub (8240S, Cell Signaling)
SMCHD1 (HPA039441, Sigma)
RING1A (09-706, Millipore)
RING1B (5694T, Cell Signaling)
CTCF (2899S, Cell Signaling)
β-tubulin (T5201, Sigma)
HNRNPK (11426-1-AP, Proteintech)
LBR (12398-1-AP, Proteintech)
RBM15 (ab70549, Abcam)
GAPDH (2118, Cell Signaling)

Validation

Most (SMCHD1, H2AK119ub, HNRNPK, LBR, and RBM15) were validated by siRNA-mediated knockdown.

Eukaryotic cell lines

Policy information about cell lines

Cell line source(s)

Female wild-type mouse embryonic fibroblast line (EY.T4) and XiΔXist female fibroblast line have been described, as referenced in the manuscript.

Authentication

Cell lines were genotyped by PCR and Sanger sequencing. Next-generation sequencing data also support the genotypes.

Mycoplasma contamination

All cell lines were tested and were found negative.

Commonly misidentified lines
(See ICLAC register)

No commonly misidentified cell lines were used.

ChIP-seq

Data deposition

☒ Confirm that both raw and final processed data have been deposited in a public database such as GEO.

☐ Confirm that you have deposited or provided access to graph files (e.g. BED files) for the called peaks.

Data access links
May remain private before publication.

https://www.ncbi.nlm.nih.gov/geo/query/acc.cgi?acc=GSE116413

Files in database submission

CWb4-1_R1.fastq.gz
CWb4-1_R2.fastq.gz
CWb4-2_R1.fastq.gz
CWb4-2_R2.fastq.gz
CWb4-3_R1.fastq.gz
CWb4-3_R2.fastq.gz
CWb4-4_R1.fastq.gz
CWb4-4_R2.fastq.gz
CWb5-1_R1.fastq.gz
CWb5-1_R2.fastq.gz
CWb5-2_R1.fastq.gz
CWb5-2_R2.fastq.gz
CWb5-3_R1.fastq.gz
CWb5-3_R2.fastq.gz
CWb5-4_R1.fastq.gz
CWb5-4_R2.fastq.gz
CWb13-2_R1.fastq.gz
CWb13-2_R2.fastq.gz
CWb17-1_R1.fastq.gz
CWb17-1_R2.fastq.gz
CWb18-1_R1.fastq.gz
CWb18-1_R2.fastq.gz
CWb24-1_R1.fastq.gz
CWb24-1_R2.fastq.gz
CWb24-2_R1.fastq.gz
CWb24-2_R2.fastq.gz
AKa7-5_R1.fastq.gz
AKa7-5_R2.fastq.gz
AKa7-6_R1.fastq.gz
AKa7-6_R2.fastq.gz
CWb26-2_R1.fastq.gz
CWb26-2_R2.fastq.gz
CWb27-1_R1.fastq.gz
CWb27-1_R2.fastq.gz
CWb27-3_R1.fastq.gz
CWb27-3_R2.fastq.gz

CWb28-1_R1.fastq.gz
CWb28-1_R2.fastq.gz
CWb28-2_R1.fastq.gz
CWb28-2_R2.fastq.gz
CWb29-1_R1.fastq.gz
CWb29-1_R2.fastq.gz
CWb29-2_R1.fastq.gz
CWb29-2_R2.fastq.gz
CWb30-1_R1.fastq.gz
CWb30-1_R2.fastq.gz
CWb30-2_R1.fastq.gz
CWb30-2_R2.fastq.gz
CWb31-1_R1.fastq.gz
CWb31-1_R2.fastq.gz
CWb31-3_R1.fastq.gz
CWb31-3_R2.fastq.gz
CWb31-4_R1.fastq.gz
CWb31-4_R2.fastq.gz
CWb31-5_R1.fastq.gz
CWb31-5_R2.fastq.gz
CWb31-6_R1.fastq.gz
CWb31-6_R2.fastq.gz
CWb32-1_R1.fastq.gz
CWb32-1_R2.fastq.gz
CWb32-2_R1.fastq.gz
CWb32-2_R2.fastq.gz
CWb32-3_R1.fastq.gz
CWb32-3_R2.fastq.gz
CWb32-4_R1.fastq.gz
CWb32-4_R2.fastq.gz
CWb32-5_R1.fastq.gz
CWb32-5_R2.fastq.gz
CWb32-6_R1.fastq.gz
CWb32-6_R2.fastq.gz
CWb33-2_R1.fastq.gz
CWb33-2_R2.fastq.gz
WEa39-1_R1.fastq.gz
WEa39-1_R2.fastq.gz
WEa39-3_R1.fastq.gz
WEa39-3_R2.fastq.gz
WEa39-4_R1.fastq.gz
WEa39-4_R2.fastq.gz
YLc2-5_R1.fastq.gz
YLc2-5_R2.fastq.gz
WT1.RNAseq.DMSO.cas.+.bw
WT1.RNAseq.DMSO.cas.-.bw
WT1.RNAseq.DMSO.mus.+.bw
WT1.RNAseq.DMSO.mus.-.bw
WT1.RNAseq.DMSO.comp.+.bw
WT1.RNAseq.DMSO.comp.-.bw
WT2.RNAseq.DMSO.cas.+.bw
WT2.RNAseq.DMSO.cas.-.bw
WT2.RNAseq.DMSO.mus.+.bw
WT2.RNAseq.DMSO.mus.-.bw
WT2.RNAseq.DMSO.comp.+.bw
WT2.RNAseq.DMSO.comp.-.bw
KO1.RNAseq.DMSO.cas.+.bw
KO1.RNAseq.DMSO.cas.-.bw
KO1.RNAseq.DMSO.mus.+.bw
KO1.RNAseq.DMSO.mus.-.bw
KO1.RNAseq.DMSO.comp.+.bw
KO1.RNAseq.DMSO.comp.-.bw
KO2.RNAseq.DMSO.cas.+.bw
KO2.RNAseq.DMSO.cas.-.bw
KO2.RNAseq.DMSO.mus.+.bw
KO2.RNAseq.DMSO.mus.-.bw
KO2.RNAseq.DMSO.comp.+.bw
KO2.RNAseq.DMSO.comp.-.bw
WT1.RNAseq.Aza.cas.+.bw
WT1.RNAseq.Aza.cas.-.bw
WT1.RNAseq.Aza.mus.+.bw
WT1.RNAseq.Aza.mus.-.bw
WT1.RNAseq.Aza.comp.+.bw
WT1.RNAseq.Aza.comp.-.bw
WT2.RNAseq.Aza.cas.+.bw

WT2.RNAseq.Aza.cas.-.bw
WT2.RNAseq.Aza.mus.+.bw
WT2.RNAseq.Aza.mus.-.bw
WT2.RNAseq.Aza.comp.+.bw
WT2.RNAseq.Aza.comp.-.bw
KO1.RNAseq.Aza.cas.+.bw
KO1.RNAseq.Aza.cas.-.bw
KO1.RNAseq.Aza.mus.+.bw
KO1.RNAseq.Aza.mus.-.bw
KO1.RNAseq.Aza.comp.+.bw
KO1.RNAseq.Aza.comp.-.bw
KO2.RNAseq.Aza.cas.+.bw
KO2.RNAseq.Aza.cas.-.bw
KO2.RNAseq.Aza.mus.+.bw
KO2.RNAseq.Aza.mus.-.bw
KO2.RNAseq.Aza.comp.+.bw
KO2.RNAseq.Aza.comp.-.bw
RNAseq_raw_read_count_table.txt
D0.ES.Xa.PC1.200kb.bedGraph
D4.EB.Xa.PC1.200kb.bedGraph
D4.EB.Xi.PC1.200kb.bedGraph
D7.EB.Xa.PC1.200kb.bedGraph
D7.EB.Xi.PC1.200kb.bedGraph
MEF.KO1.Xa.PC1.100kb.bedGraph
MEF.KO1.Xi.PC1.100kb.bedGraph
MEF.WT1.Xa.PC1.100kb.bedGraph
MEF.WT1.Xi.PC1.100kb.bedGraph
MEF.X_chromosome.insulation_score.txt
delta.MEF.ChIP.H2AK119ub.comp.bw
delta.MEF.ChIP.H3K27me3.rep1.comp.bw
delta.MEF.ChIP.H3K27me3.rep2.comp.bw
KO.MEF.CHART.input.rep1.cas.bw
KO.MEF.CHART.input.rep1.comp.bw
KO.MEF.CHART.input.rep1.mus.bw
KO.MEF.CHART.input.rep2.cas.bw
KO.MEF.CHART.input.rep2.comp.bw
KO.MEF.CHART.input.rep2.mus.bw
KO.MEF.CHART.Xist.rep1.cas.bw
KO.MEF.CHART.Xist.rep1.comp.bw
KO.MEF.CHART.Xist.rep1.mus.bw
KO.MEF.CHART.Xist.rep2.cas.bw
KO.MEF.CHART.Xist.rep2.comp.bw
KO.MEF.CHART.Xist.rep2.mus.bw
KO.MEF.ChIP.H2AK119ub.cas.bw
KO.MEF.ChIP.H2AK119ub.comp.bw
KO.MEF.ChIP.H2AK119ub.mus.bw
KO.MEF.ChIP.H3K27me3.rep1.cas.bw
KO.MEF.ChIP.H3K27me3.rep1.comp.bw
KO.MEF.ChIP.H3K27me3.rep1.mus.bw
KO.MEF.ChIP.H3K27me3.rep2.cas.bw
KO.MEF.ChIP.H3K27me3.rep2.comp.bw
KO.MEF.ChIP.H3K27me3.rep2.mus.bw
KO.MEF.ChIP.input.cas.bw
KO.MEF.ChIP.input.comp.bw
KO.MEF.ChIP.input.mus.bw
SPP.normalized.delta.MEF.CHART.Xist.rep1.comp.bw
SPP.normalized.delta.MEF.CHART.Xist.rep2.comp.bw
SPP.normalized.KO.MEF.CHART.Xist.rep1.comp.bw
SPP.normalized.KO.MEF.CHART.Xist.rep2.comp.bw
SPP.normalized.WT.MEF.CHART.Xist.rep1.comp.bw
SPP.normalized.WT.MEF.CHART.Xist.rep2.comp.bw
WT.MEF.CHART.input.rep1.cas.bw
WT.MEF.CHART.input.rep1.comp.bw
WT.MEF.CHART.input.rep1.mus.bw
WT.MEF.CHART.input.rep2.cas.bw
WT.MEF.CHART.input.rep2.comp.bw
WT.MEF.CHART.input.rep2.mus.bw
WT.MEF.CHART.Xist.rep1.cas.bw
WT.MEF.CHART.Xist.rep1.comp.bw
WT.MEF.CHART.Xist.rep1.mus.bw
WT.MEF.CHART.Xist.rep2.cas.bw
WT.MEF.CHART.Xist.rep2.comp.bw
WT.MEF.CHART.Xist.rep2.mus.bw
WT.MEF.ChIP.H2AK119ub.cas.bw
WT.MEF.ChIP.H2AK119ub.comp.bw

WT.MEF.ChIP.H2AK119ub.mus.bw
WT.MEF.ChIP.H3K27me3.rep1.cas.bw
WT.MEF.ChIP.H3K27me3.rep1.comp.bw
WT.MEF.ChIP.H3K27me3.rep1.mus.bw
WT.MEF.ChIP.H3K27me3.rep2.cas.bw
WT.MEF.ChIP.H3K27me3.rep2.comp.bw
WT.MEF.ChIP.H3K27me3.rep2.mus.bw
WT.MEF.ChIP.input.cas.bw
WT.MEF.ChIP.input.comp.bw
WT.MEF.ChIP.input.mus.bw
Downsampled.KO.MEF.HNRNPK_KD.Hi-C.rep1.cas.summary.txt
Downsampled.KO.MEF.HNRNPK_KD.Hi-C.rep1.mus.summary.txt
Downsampled.KO.MEF.HNRNPK_KD.Hi-C.rep2.cas.summary.txt
Downsampled.KO.MEF.HNRNPK_KD.Hi-C.rep2.mus.summary.txt
Downsampled.KO.MEF.No_treatment.Hi-C.cas.summary.txt
Downsampled.KO.MEF.No_treatment.Hi-C.mus.summary.txt
Downsampled.KO.MEF.PRC1_KD.Hi-C.rep1.cas.summary.txt
Downsampled.KO.MEF.PRC1_KD.Hi-C.rep1.mus.summary.txt
Downsampled.KO.MEF.PRC1_KD.Hi-C.rep2.cas.summary.txt
Downsampled.KO.MEF.PRC1_KD.Hi-C.rep2.mus.summary.txt
Downsampled.KO.MEF.Scramble.Hi-C.rep1.cas.summary.txt
Downsampled.KO.MEF.Scramble.Hi-C.rep1.mus.summary.txt
Downsampled.KO.MEF.Scramble.Hi-C.rep2.cas.summary.txt
Downsampled.KO.MEF.Scramble.Hi-C.rep2.mus.summary.txt
Downsampled.WT.MEF.HNRNPK_KD.Hi-C.rep1.cas.summary.txt
Downsampled.WT.MEF.HNRNPK_KD.Hi-C.rep1.mus.summary.txt
Downsampled.WT.MEF.HNRNPK_KD.Hi-C.rep2.cas.summary.txt
Downsampled.WT.MEF.HNRNPK_KD.Hi-C.rep2.mus.summary.txt
Downsampled.WT.MEF.No_treatment.Hi-C.cas.summary.txt
Downsampled.WT.MEF.No_treatment.Hi-C.mus.summary.txt
Downsampled.WT.MEF.PRC1_KD.Hi-C.cas.summary.txt
Downsampled.WT.MEF.PRC1_KD.Hi-C.mus.summary.txt
Downsampled.WT.MEF.Scramble.Hi-C.cas.summary.txt
Downsampled.WT.MEF.Scramble.Hi-C.mus.summary.txt
Downsampled.Xist.deletion.fibroblast.Hi-C.cas.summary.txt
Downsampled.Xist.deletion.fibroblast.Hi-C.mus.summary.txt
KO.MEF.HNRNPK_KD.Hi-C.rep1.cas.summary.txt
KO.MEF.HNRNPK_KD.Hi-C.rep1.comp.summary.txt
KO.MEF.HNRNPK_KD.Hi-C.rep1.mus.summary.txt
KO.MEF.HNRNPK_KD.Hi-C.rep2.cas.summary.txt
KO.MEF.HNRNPK_KD.Hi-C.rep2.comp.summary.txt
KO.MEF.HNRNPK_KD.Hi-C.rep2.mus.summary.txt
KO.MEF.No_treatment.Hi-C.cas.summary.txt
KO.MEF.No_treatment.Hi-C.comp.summary.txt
KO.MEF.No_treatment.Hi-C.mus.summary.txt
KO.MEF.PRC1_KD.Hi-C.rep1.cas.summary.txt
KO.MEF.PRC1_KD.Hi-C.rep1.comp.summary.txt
KO.MEF.PRC1_KD.Hi-C.rep1.mus.summary.txt
KO.MEF.PRC1_KD.Hi-C.rep2.cas.summary.txt
KO.MEF.PRC1_KD.Hi-C.rep2.comp.summary.txt
KO.MEF.PRC1_KD.Hi-C.rep2.mus.summary.txt
KO.MEF.Scramble.Hi-C.rep1.cas.summary.txt
KO.MEF.Scramble.Hi-C.rep1.comp.summary.txt
KO.MEF.Scramble.Hi-C.rep1.mus.summary.txt
KO.MEF.Scramble.Hi-C.rep2.cas.summary.txt
KO.MEF.Scramble.Hi-C.rep2.comp.summary.txt
KO.MEF.Scramble.Hi-C.rep2.mus.summary.txt
WT.MEF.HNRNPK_KD.Hi-C.rep1.cas.summary.txt
WT.MEF.HNRNPK_KD.Hi-C.rep1.comp.summary.txt
WT.MEF.HNRNPK_KD.Hi-C.rep1.mus.summary.txt
WT.MEF.HNRNPK_KD.Hi-C.rep2.cas.summary.txt
WT.MEF.HNRNPK_KD.Hi-C.rep2.comp.summary.txt
WT.MEF.HNRNPK_KD.Hi-C.rep2.mus.summary.txt
WT.MEF.No_treatment.Hi-C.cas.summary.txt
WT.MEF.No_treatment.Hi-C.comp.summary.txt
WT.MEF.No_treatment.Hi-C.mus.summary.txt
WT.MEF.PRC1_KD.Hi-C.cas.summary.txt
WT.MEF.PRC1_KD.Hi-C.comp.summary.txt
WT.MEF.PRC1_KD.Hi-C.mus.summary.txt
WT.MEF.Scramble.Hi-C.cas.summary.txt
WT.MEF.Scramble.Hi-C.comp.summary.txt
WT.MEF.Scramble.Hi-C.mus.summary.txt
Xist.deletion.fibroblast.Hi-C.cas.summary.txt
Xist.deletion.fibroblast.Hi-C.comp.summary.txt
Xist.deletion.fibroblast.Hi-C.mus.summary.txt

KO.MEF.HNRNPK_KD.Hi-C.rep1.Xa.PC1.200kb.bedGraph
KO.MEF.HNRNPK_KD.Hi-C.rep1.Xa.PC2.200kb.bedGraph
KO.MEF.HNRNPK_KD.Hi-C.rep1.Xi.PC1.200kb.bedGraph
KO.MEF.HNRNPK_KD.Hi-C.rep1.Xi.PC2.200kb.bedGraph
KO.MEF.HNRNPK_KD.Hi-C.rep2.Xa.PC1.200kb.bedGraph
KO.MEF.HNRNPK_KD.Hi-C.rep2.Xa.PC2.200kb.bedGraph
KO.MEF.HNRNPK_KD.Hi-C.rep2.Xi.PC1.200kb.bedGraph
KO.MEF.HNRNPK_KD.Hi-C.rep2.Xi.PC2.200kb.bedGraph
KO.MEF.No_treatment.Hi-C.Xa.PC1.200kb.bedGraph
KO.MEF.No_treatment.Hi-C.Xa.PC2.200kb.bedGraph
KO.MEF.No_treatment.Hi-C.Xi.PC1.200kb.bedGraph
KO.MEF.No_treatment.Hi-C.Xi.PC2.200kb.bedGraph
KO.MEF.PRC1_KD.Hi-C.rep1.Xa.PC1.200kb.bedGraph
KO.MEF.PRC1_KD.Hi-C.rep1.Xa.PC2.200kb.bedGraph
KO.MEF.PRC1_KD.Hi-C.rep1.Xi.PC1.200kb.bedGraph
KO.MEF.PRC1_KD.Hi-C.rep1.Xi.PC2.200kb.bedGraph
KO.MEF.PRC1_KD.Hi-C.rep2.Xa.PC1.200kb.bedGraph
KO.MEF.PRC1_KD.Hi-C.rep2.Xa.PC2.200kb.bedGraph
KO.MEF.PRC1_KD.Hi-C.rep2.Xi.PC1.200kb.bedGraph
KO.MEF.PRC1_KD.Hi-C.rep2.Xi.PC2.200kb.bedGraph
KO.MEF.Scramble.Hi-C.rep1.Xa.PC1.200kb.bedGraph
KO.MEF.Scramble.Hi-C.rep1.Xa.PC2.200kb.bedGraph
KO.MEF.Scramble.Hi-C.rep1.Xi.PC1.200kb.bedGraph
KO.MEF.Scramble.Hi-C.rep1.Xi.PC2.200kb.bedGraph
KO.MEF.Scramble.Hi-C.rep2.Xa.PC1.200kb.bedGraph
KO.MEF.Scramble.Hi-C.rep2.Xa.PC2.200kb.bedGraph
KO.MEF.Scramble.Hi-C.rep2.Xi.PC1.200kb.bedGraph
KO.MEF.Scramble.Hi-C.rep2.Xi.PC2.200kb.bedGraph
WT.MEF.HNRNPK_KD.Hi-C.rep1.Xa.PC1.200kb.bedGraph
WT.MEF.HNRNPK_KD.Hi-C.rep1.Xa.PC2.200kb.bedGraph
WT.MEF.HNRNPK_KD.Hi-C.rep1.Xi.PC1.200kb.bedGraph
WT.MEF.HNRNPK_KD.Hi-C.rep1.Xi.PC2.200kb.bedGraph
WT.MEF.HNRNPK_KD.Hi-C.rep2.Xa.PC1.200kb.bedGraph
WT.MEF.HNRNPK_KD.Hi-C.rep2.Xa.PC2.200kb.bedGraph
WT.MEF.HNRNPK_KD.Hi-C.rep2.Xi.PC1.200kb.bedGraph
WT.MEF.HNRNPK_KD.Hi-C.rep2.Xi.PC2.200kb.bedGraph
WT.MEF.No_treatment.Hi-C.Xa.PC1.200kb.bedGraph
WT.MEF.No_treatment.Hi-C.Xa.PC2.200kb.bedGraph
WT.MEF.No_treatment.Hi-C.Xi.PC1.200kb.bedGraph
WT.MEF.No_treatment.Hi-C.Xi.PC2.200kb.bedGraph
WT.MEF.PRC1_KD.Hi-C.Xa.PC1.200kb.bedGraph
WT.MEF.PRC1_KD.Hi-C.Xa.PC2.200kb.bedGraph
WT.MEF.PRC1_KD.Hi-C.Xi.PC1.200kb.bedGraph
WT.MEF.PRC1_KD.Hi-C.Xi.PC2.200kb.bedGraph
WT.MEF.Scramble.Hi-C.Xa.PC1.200kb.bedGraph
WT.MEF.Scramble.Hi-C.Xa.PC2.200kb.bedGraph
WT.MEF.Scramble.Hi-C.Xi.PC1.200kb.bedGraph
WT.MEF.Scramble.Hi-C.Xi.PC2.200kb.bedGraph
Xist.deletion.fibroblast.Hi-C.Xa.PC1.200kb.bedGraph
Xist.deletion.fibroblast.Hi-C.Xa.PC2.200kb.bedGraph
Xist.deletion.fibroblast.Hi-C.Xi.PC1.200kb.bedGraph
Xist.deletion.fibroblast.Hi-C.Xi.PC2.200kb.bedGraph

Genome browser session
(e.g. UCSC)

Not applicable.

Methodology

Replicates

H3K27me3 ChIP-seq was performed in two biological replicate. H2AK119ub ChIP-seq was performed in one biological replicate.

Sequencing depth

~25 million paired end reads were generated for each library.

Antibodies

anti-H3K27me3 (07-449, Millipore); anti-H2AK119ub (8240, Cell Signaling)

Peak calling parameters

We did not perform peak calling as H3K27me3 and H2AK119ub forms broad domains covering almost the entire inactive X chromosome.

Data quality

We performed allele-specific alignment, which showed that H3K27me3 and H2AK119ub ChIP signals originate predominantly from the inactive X chromosome, consistent with immunostaining showing that these marks are enriched on the inactive X chromosome.

Software

The software used was described in the "Methods" and was also listed below:

Software	Trim Galore!<br>novoalign/novoalign_v3-1.00.02<br>samtools0118
